# From Fragile to Firm: Reinforcement of Excess Electron Binding in Dipole-Bound Anions through Sigma-Hole and Hydrogen-Bond Interactions

**DOI:** 10.1021/acs.jpca.5c05103

**Published:** 2025-09-02

**Authors:** Piotr Skurski, Jakub Brzeski

**Affiliations:** † Faculty of Chemistry, 49646 University of Gdańsk, Wita Stwosza 63, 80-308 Gdańsk, Poland; ‡ QSAR Lab Ltd., Trzy Lipy 3, 80-172 Gdańsk, Poland; § Department of Chemistry, University of Utah, Salt Lake City, Utah 84112, United States

## Abstract

The stability of dipole-bound anions formed by small polar molecules and their noncovalent complexes was investigated using highly correlated *ab initio* methods and flexible basis sets. The HCN, HNC, and ClCN species were found to form weakly bound anions of dipole-bound nature, with excess electron binding energies not exceeding 50 cm^–1^. When ClCN forms noncovalent complexes with either HCN or HNC, two isomeric structures become possible, stabilized either by a hydrogen bond or by a σ-hole interaction. All noncovalent complexes exhibit dipole moments increased by more than a factor of 2 compared to the isolated components, which in turn facilitates significantly stronger excess electron binding. Among them, hydrogen-bonded complexes display stronger intermolecular interactions and, consequently, substantially enhanced binding of the excess electron. The predicted excess electron binding energies span from 413 to 1265 cm^–1^ for the four resulting dipole-bound anions, namely, (HCN···ClCN)^−^, (ClCN···HCN)^−^, (HNC···ClCN)^−^, and (ClCN···HNC)^−^, with hydrogen-bonded species being approximately twice as strongly electronically bound as their σ-hole-stabilized counterparts. These results demonstrate that excess electron binding can increase by an order of magnitude upon the formation of a noncovalent complex, even when its individual components exhibit only marginal ability to accommodate an excess electron.

## Graphical abstract

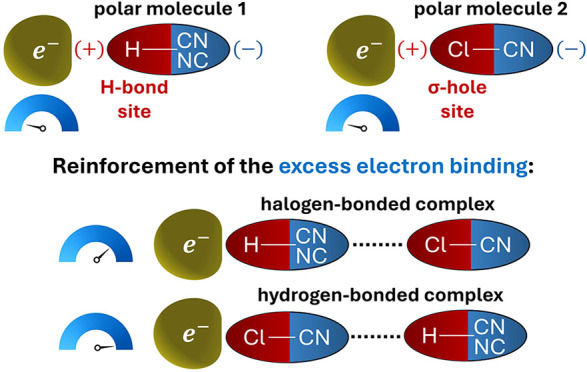

## 1 Introduction

Dipole-bound anionic states are typically classified as such when their existence is dictated by the long-range dipole potential generated by the neutral parent molecule.^1^ However, although the dipole interaction plays a major role in binding the excess electron, none of these anions is purely or exclusively dipole-bound.^2^ This is because the excess electron binding energy (*D*) depends not only on the dipole moment (μ), but also on the characteristics of the molecule’s occupied orbitals, as reflected in the associated Coulomb and exchange potentials. In fact, it has been shown that the electron-dipole attraction and other charge-multipole interactions, combined with Coulomb and exchange effects, often account for less than half of the total excess electron binding energy in such systems.^1,3,4^ The remaining contribution typically arises from electron correlation effects (most notably, dispersion interactions^1,5,6^ ). Therefore, for real molecular systems, one can conclude that there are no anions that are entirely dipole-bound, even though the charge-dipole interaction is the component that attracts the excess electron at the longest range. Despite the terminological subtleties highlighted here, a typical dipole-bound anion is bound due to the static Coulomb interaction with the charge distribution of the neutral molecule, and the excess electron in such an anion occupies a fully symmetric orbital (that is, one transforming according to the totally symmetric irreducible representation of the relevant point group), primarily localized on the positive side of the molecular dipole and outside the range of the valence orbitals. This reflects the requirement that the charge distribution of the excess electron be consistent with the electrostatic potential generated by the lowest nonvanishing multipole moment (namely the dipole moment) of the parent neutral molecule. Importantly, this type of orbital arises from the presence of a long-range attractive dipole potential, but it also experiences shorter-range attractive and repulsive valence interactions (see the comprehensive discussion in ref 7).

Over the past few decades, a large number of dipole-bound anions have been characterized in the literature through both experimental^8–22^ and theoretical studies.^1,3–6,23–34^ These include anions formed by small molecules such as HCN,^23^ CH_3_CN,^3^ or HPPH_3_,^24^ as well as by somewhat larger, biologically relevant species such as uracil^13,14,27,28^ and adenine,^29^ and even by molecular complexes stabilized through hydrogen bonding.^1,15,16,25,26,35^ Particularly interesting among dipole-bound anions are those complexes in which intermolecular interactions enhance excess electron binding - and in some cases, even make the binding of the excess electron possible. Most of these systems rely on hydrogen bonding, but examples are also known in which the integrity of the complex and the resulting polarity (sufficient to support dipole binding) arise from charge-transfer interactions.^36,37^

Despite numerous reports on dipole-bound anions published thus far, to the best of our knowledge, none has addressed a situation in which excess electron binding in such systems is reinforced by a σ-hole interaction. This observation provided the direct motivation for our investigation. Hence, in this contribution, we present results obtained using correlated *ab initio* methods for anionic complexes formed by HCN or HNC with ClCN. As we show further, both hydrogen cyanide and its tautomer, hydrogen isocyanide, form two distinct isomers with the cyanogen chloride molecule: one stabilized by a hydrogen bond, and the other stabilized through halogen bonding (which is a subclass of σ-hole interactions).^38^ Since each of these complexes is capable of excess electron binding due to its dipole potential, they offer a convenient model for quantifying the effect that each of these noncovalent interactions has on the resulting excess electron binding energy in the corresponding anionic systems.

## 2 Methods

The equilibrium structures of closed-shell neutral systems, HCN, HNC, ClCN, HCN···ClCN, ClCN···HCN, HNC···ClCN, and ClCN···HNC, as well as their corresponding daughter anions, were determined using the coupled-cluster method with single and double excitations (CCSD),^39,40^ correlating all core and valence orbitals. All calculations employed the aug-cc-pVTZ basis set,^41,42^ supplemented by an additional set of diffuse functions centered at the positive end of the molecular dipole (that is, on the Cl atom for ClCN, ClCN···HCN, and ClCN···HCN, or on the H atom in all other cases). These extra diffuse functions did not share exponent values and were constructed as even-tempered sets,^43^ consisting of eight *s*, nine *p*, and four *d* functions. The geometric progression ratio was equal to 3.2.^44,45^ For each angular momentum, we started to build up the exponents of the extra diffuse functions from the smallest exponent of the same symmetry present in the original aug-cc-pVTZ basis set, defined for either H (for HCN, HNC, HCN···ClCN, and HNC···ClCN) or Cl (for ClCN, ClCN···HCN, and ClCN···HNC). The lowest exponents achieved were 2.297383616 × 10^–6^ (*s*), 2.899014361 × 10^–6^ (*p*), and 2.355575562 × 10^–3^ (*d*) a.u. for the H-centered diffuse sets, and 5.375113687 × 10^–6^ (*s*), 1.190869625 × 10^–6^ (*p*), and 1.287460327 × 10^–3^ (*d*) a.u. for the Cl-centered ones. In each case, the lowest eigenvalue of the atomic orbital overlap matrix was examined to ensure that near-linear dependency was not an issue.

As (HCN)^−^, (HNC)^−^, (ClCN)^−^, (HCN···ClCN)^−^, (ClCN···HCN)^−^, (HNC···ClCN)^−^, and (ClCN···HNC)^−^ are open-shell species, we employed methods based on an unrestricted Hartree–Fock (UHF) reference. To ensure that minimal spin contamination entered the final wave functions, the expectation value ⟨*S* ^2^⟩ was evaluated for each doublet anion, yielding values not exceeding 0.75001 at the UHF level. This confirms that spin contamination is negligible and does not significantly affect our results.

Harmonic vibrational frequencies were computed at the same CCSD­(full)/aug-cc-pVTZ+8*s*9*p*4*d* level of theory to confirm that all optimized structures correspond to true minima on the potential energy surface.

Excess electron binding energies (*D*) were calculated using a supermolecular approach, defined as the difference between the total energies of the neutral species (*E_N_*) and their anions (*E_A_*). To ensure size extensivity, the electron binding energies were evaluated using Møller–Plesset perturbation theory^46–48^ up to the fourth order (MP2, MP3, MP4), as well as the coupled-cluster method with single, double, and noniterative triple excitations (CCSD­(T)),^49^ in all cases correlating all electrons and employing the aug-cc-pVTZ+8*s*9*p*4*d* basis set. Additionally, *D* was analyzed within a perturbative framework specifically developed for dipole-bound anions.^6^

The simplest theoretical estimate of *D* follows from Koopmans’ theorem (KT),^50^ where the excess electron binding energy *D* ^*KT*^ is approximated as the negative of the energy of the relevant unoccupied orbital obtained from a Hartree–Fock self-consistent-field (SCF) calculation on the neutral molecule. This approximation neglects both orbital relaxation and electron correlation effects. These effects were accounted for by performing SCF and CCSD­(T) calculations on both the neutral and anionic systems. The polarization of the neutral molecular host (*N*) by the excess electron, as well as the back-polarization of the electron by the host, were captured by performing the SCF calculation for the anion (*A*). The corresponding induction effects on *D* are given by Δ*D*_*ind*_^*SCF*^ = *D* ^*SCF*^ – *D* ^*KT*^, where *D* ^*SCF*^ = *E*_*N*_^*SCF*^ – *E*_*A*_^*SCF*^. The second-order Møller–Plesset (MP2) contribution to the electron binding energy (Δ*D* ^*MP*2^ = *D* ^*MP*2^ – *D* ^*SCF*^) was decomposed into a dispersion-like interaction between the loosely bound electron (*lbe*) and the electrons of the neutral molecule, denoted Δ*D*_*disp*_^*MP*2^, and a nondispersion term, Δ*D*_*no‑disp*_^*MP*2^. The Δ*D*_*disp*_^*MP*2^ component was evaluated as a sum over all orbital pair contributions *e*_*lbe,i*_, corresponding to excitations of the form ϕ_*lbe*_ϕ_*i*_ → ϕ_*v*_ϕ_*v*′_, where ϕ_*lbe*_ is the orbital occupied by the loosely bound electron, ϕ_*i*_ is one of the other occupied orbitals of the host, and ϕ_*v*_ and ϕ_*v*′_ are unoccupied virtual orbitals in the zeroth-order wave function.^6^ The nondispersion component (that is, the Δ*D*_*no‑disp*_^*MP*2^ term) primarily accounts for electron correlation corrections to the static Coulomb interaction between the excess electron and the charge distribution of the neutral host. Higher-order MP contributions to *D* were defined recursively as Δ*D* ^*MPn*^ = *D* ^*MPn*^ – *D* ^*MP*(*n*–1)^ (for *n* = 3, 4), while contributions beyond the fourth order were approximated by the difference between CCSD­(T) and MP4 results: Δ*D* ^*CCSD*(*T*)^ = *D* ^*CCSD*(*T*)^ – *D* ^*MP*4^.

Additionally, symmetry-adapted perturbation theory (SAPT) was used to evaluate the intermolecular interaction energies in the studied complexes and to decompose them into physically meaningful components.^51^ SAPT calculations were carried out at two levels of theory: SAPT0 and SAPT2 + 3­(CCD)­δ_MP2_.^52–54^ The SAPT0 method was applied to all noncovalently bound systems (both neutral and anionic), while the higher-level SAPT2 + 3­(CCD)­δ_MP2_ – which includes coupled-cluster correction to dispersion and δ_MP2_ corrections accounting for higher-order induction effects – was used only for closed-shell systems. This limitation arises because SAPT0 is the only available SAPT method capable of handling open-shell species.^55^ For SAPT0 calculations on anionic systems, the unpaired electron was assigned to the molecular fragment bearing the additional diffuse basis set, that is, the positive end of the dipole. All SAPT calculations were performed using the aug-cc-pVTZ+8*s*9*p*4*d* basis set.

The SAPT calculations were carried out with the PSI4 software package (version 1.9.1),^56^ while all remaining *ab initio* calculations were performed using GAUSSIAN16 (Rev.C.01).^57^

## 3 Results

### 3.1 The (HCN)^−^, (HNC)^−^, and (ClCN)^−^ Dipole-Bound Anions

The ability of hydrogen cyanide and its tautomer, hydrogen isocyanide, to form anionic states has already been investigated and described in the literature,^4,23,58^ in contrast to the case of cyanogen chloride, for which the possibility of forming stable anions has not been previously addressed. It should be noted that the earlier results obtained for the (HCN)^−^ and (HNC)^−^ anions were considered reliable, primarily due to the use of advanced *ab initio* methods (QCISD, CCSD­(T)) in combination with extended basis sets (aug-cc-pV*n*Z, *n* = *D*, *T*, augmented with 8*s*9*p*4*d* functions).^4,23^ Nevertheless, in the present study we decided to recalculate the HCN/(HCN)^−^ and HNC/(HNC)^−^ systems, both in terms of geometry optimization and excess electron binding energy determination. This was motivated, first, by the need to obtain results at the same level of theory as those for the other systems analyzed in this work, and second, by the fact that the theoretical approach adopted here – CCSD-based gradient and force constant calculations with full electron correlation (including core orbitals) during both geometry optimization and binding energy estimation – is in fact more accurate than the theoretical frameworks used in previous studies.

As shown by our calculations, none of the HCN, HNC, or ClCN systems forms a valence-bound anion. However, the dipole moments calculated for these neutral molecules exceed 2.5 D, which suggests the possibility of binding an extra electron via the dipole potential to form stable dipole-bound anionic states.^1,2^ Our computations confirm that such anionic states can indeed be formed by these systems. Before discussing in detail the electronic stability of the (HCN)^−^, (HNC)^−^, and (ClCN)^−^ anions, we first address structural aspects, specifically the geometry relaxation that follows excess electron attachment. The geometrical parameters collected in Table 1 for the neutral and anionic forms of the three molecules show that all of them are linear, corresponding to C_∞v_ symmetry. In addition, the geometry relaxation observed upon anion formation is negligibly small. The bond lengths reported in Table 1 demonstrate that the attachment of an excess electron to any of the HCN, HNC, or ClCN molecules leads to only a marginal change (not exceeding 0.001 Å) in individual bond lengths. Such minimal structural relaxation upon excess electron attachment, although typical for many previously reported dipole-bound anionic states,^1^ suggests that the excess electron binding energies for the studied systems are likely to be relatively low. Indeed, as we are about to demonstrate, the (HCN)^−^, (HNC)^−^, and (ClCN)^−^ anions are very weakly electronically bound systems, with excess electron binding energies in the range of 10–46 cm^–1^ (1–6 meV).

**Table 1:** Structural Parameters and Harmonic Vibrational Frequencies of Neutral and Anionic C_∞v_-Symmetry Stationary Point Structures of HCN, HNC, ClCN^a^

HCN/(HCN)^−^	
HCN	(HCN)^−^
*r*(H–C) = 1.057; *r*(C–N) = 1.148	*r*(H–C) = 1.057; *r*(C–N) = 1.148
ν_1,2_(π) = 724^b^; ν(σ) = 2197^c^; ν(σ) = 3511^d^	ν_1,2_(π) = 724^b^; ν(σ) = 2198^c^; ν(σ) = 3510^d^

HNC/(HNC)^−^	
HNC	(HNC)^−^
*r*(H–N) = 0.992; *r*(N–C) = 1.164	*r*(H–N) = 0.992; *r*(N–C) = 1.164
ν_1,2_(π) = 501^b^; ν(σ) = 2131^c^; ν(σ) = 3879^d^	ν_1,2_(π) = 616^b^; ν(σ) = 2132^c^; ν(σ) = 3866^d^

ClCN/(ClCN)^−^	
ClCN	(ClCN)^−^
*r*(Cl–C) = 1.634; *r*(C–N) = 1.152	*r*(Cl–C) = 1.635; *r*(C–N) = 1.152
ν_1,2_(π) = 394^b^; ν(σ) = 759^c^; ν(σ) = 234^d^	ν_1,2_(π) = 370^b^; ν(σ) = 750^c^; ν(σ) = 2338^d^

a Bond lengths *r* are given in Å, and vibrational frequencies ν are provided in cm^–1^. All results were obtained at the CCSD­(full)/aug-cc-pVTZ+8*s*9*p*4*d* level of theory.

b Bending.

c In-phase stretching.

d Out-of-phase stretching.

As mentioned in the preceding paragraph, each of the neutral molecules HCN, HNC, and ClCN is sufficiently polar to bind an excess electron via the dipole potential. This is confirmed by the dipole moment values presented in Table 2, which refer to the neutral species but were calculated for two geometries: that of the neutral molecule and that of the corresponding anion. The dipole moments, obtained using both uncorrelated (μ^*SCF*^) and correlated (μ^*MP2*^, μ^*CCSD*^), electron densities, exceed 2.88*D* in all cases. Accordingly, it is not surprising that the excess electron in each of these systems is bound already at the electrostatic-exchange level (KT). It should be noted, however, that the stability of these anions at the KT level, resulting from electrostatic and exchange interactions of the extra electron with the SCF charge distribution of the neutral molecule, is extremely low. Specifically, the Koopmans’ theorem electron binding energy (*D* ^*KT*^) amounts to only 11 cm^–1^ for (HCN)^−^ and 3 cm^–1^ for both (HNC)^−^ and (ClCN)^−^, which correlates well with the corresponding μ^*SCF*^ values of the neutral species (see Table 2).

**Table 2:** Incremental Excess Electron Binding Energies (in cm^–1^) for the Anionic States of HCN, HNC, and ClCN Molecules, Obtained Using Neutral and Anionic Geometries Optimized at the CCSD­(full)/aug-cc-pVTZ+8*s*9*p*4*d* Theory Level^a^

	(HCN)^−^ neutral geometry	(HCN)^−^ anionic geometry	(HNC)^−^ neutral geometry	(HNC)^−^ anionic geometry	(ClCN)^−^ neutral geometry	(ClCN)^−^ anionic geometry
*D* ^*KT*^	11	11	3	3	3	3
Δ*D*_*ind*_^*SCF*^	0	0	0	1	1	1
Δ*D*_*disp*_^*MP*2^	11	11	5	5	7	7
Δ*D*_*no‑disp*_^*MP*2^	–10	–10	6	6	–3	–3
Δ*D* ^*MP*3^	0	0	–3	–4	0	–1
Δ*D* ^*MP*4^	1	1	3	3	2	3
Δ*D* ^*CCSD*(*T*)^	–3	–3	32	32	12	12
sum (total *D*)	10	10	46	46	22	22
μ^*SCF*^	3.279	3.279	2.887	2.890	3.070	3.070
μ^*MP*2^	3.026	3.026	3.286	3.288	2.895	2.895
μ^*CCSD*^	3.043	3.043	3.101	3.103	2.896	2.896

a Dipole moments (in Debyes) of the neutral systems, evaluated at both neutral and anionic geometries, were calculated using SCF (μ*^SCF^*), MP2 (μ*^MP2^*), and CCSD (μ*^CCSD^*) electron densities.

Before proceeding with a discussion of the contributions to *D* in the cases of the (HCN)^−^, (HNC)^−^, and (ClCN)^−^ anions we wish to note that, due to the negligibly small geometry relaxation upon excess electron attachment in each of these systems (see Table 1), it is not necessary to separately consider the results obtained for the neutral and anionic geometries. This is also evident from the values of the partial contributions to the total *D* listed in Table 2, which are nearly identical (within 1 cm^–1^) for each pair of geometries corresponding to a given anion. Therefore, in the following discussion concerning these three structurally simple anions, we will focus on the values obtained for the equilibrium anionic structures.

As shown in Table 2, the electron binding energy *D* was partitioned into incremental contributions calculated at increasing levels of theory: KT, SCF, MP*n* (*n* = 2, 3, 4), and CCSD­(T). One of the subsequent contributions to *D* is the induction term, Δ*D*_*ind*_^*SCF*^, which accounts for orbital relaxation and thus includes both the static polarization of the neutral molecule by the excess electron and the secondary effect of back-polarization. However, the Δ*D*_*ind*_^*SCF*^ values listed in Table 2 are very small (not exceeding 1 cm^–1^), indicating that the corresponding effects are nearly negligible.

The contribution denoted Δ*D*_*disp*_^*MP*2^ arises from dynamical correlation between the excess electron and the electrons of the neutral molecule. This stabilizing effect, caused by quantum mechanical charge fluctuations, is approximately twice as large as *D* ^*KT*^ for (HNC)^−^ and (ClCN)^−^, and equal to *D* ^*KT*^ for (HCN)^−^, which is consistent with earlier results reported for many other dipole-bound anions, particularly those exhibiting relatively small total excess electron binding energies.^5,6^

In addition to the dispersion interaction, electron correlation also affects the charge distribution of the neutral molecule (and thus its dipole moment), which in turn influences the electrostatic interaction with the excess electron. This effect appears for the first time at the second-order Møller–Plesset level, where it is captured by the Δ*D*_*no‑disp*_^*MP*2^. The Δ*D*_*no‑disp*_^*MP*2^ values are strongly destabilizing for the (HCN)^−^ and (ClCN)^−^ anions, nearly canceling the stabilization predicted at the KT level, as in these cases the Δ*D*_*no‑disp*_^*MP*2^ term is nearly equal in magnitude but opposite in sign to the corresponding *D* ^*KT*^ value. The destabilizing nature of the Δ*D*_*no‑disp*_^*MP*2^ contribution is consistent with the fact that electron correlation effects (approximated at the MP2 level) reduce the dipole moment of the neutral HCN and ClCN molecules by 0.253 and 0.175 D, respectively, compared to the μ^*SCF*^ value (see Table 2). In contrast, for the (HNC)^−^ anion, the Δ*D*_*no‑disp*_^*MP*2^ term is stabilizing and twice as large as *D* ^*KT*^. This clearly indicates that, in the case of the neutral HNC molecule, the charge distribution at the SCF (uncorrelated) level is inadequate in the sense that the molecular polarity is underestimated. This conclusion is further supported by the observation that the inclusion of electron correlation effects at the MP2 level increases the dipole moment of the neutral HNC molecule by nearly 0.4*D* (see Table 2). It is also worth noting that corrections beyond second-order change the dipole moments of HCN, and ClCN only slightly (by less than 0.017 D) compared to the values obtained from the MP2 electron densities, whereas for HNC this change is significant and involves a reduction of the dipole moment by 0.185*D* (cf. μ^*CCSD*^ and μ^*MP*2^ values in Table 2).

An analysis of the contributions to *D* obtained at the MP*n* level (*n* = 2, 3, 4) shows that one cannot rely on the convergence of the Møller–Plesset series for the electron binding energies of the (HCN)^−^, (HNC)^−^, and (ClCN)^−^ anions, as Table 2 affirms. The Δ*D* ^*MP*3^ contributions are either close to zero (for (HCN)^−^ and (ClCN)^−^) or destabilizing (for (HNC)^−^), whereas the Δ*D* ^*MP*4^ terms are stabilizing and are either comparable in magnitude to *D* ^*KT*^ (for (HNC)^−^ and (ClCN)^−^), or relatively small yet similar to the total Δ*D* ^*MP*2^ contribution (Δ*D*_*disp*_^*MP*2^ + Δ*D*_*no‑disp*_^*MP*2^) in the case of (HCN)^−^. For this reason, it was necessary to estimate higher-order electron correlation effects, which were approximated by Δ*D* ^*CCSD*(*T*)^. The Δ*D* ^*CCSD*(*T*)^ values collected in Table 2 show that these effects are substantial and stabilizing in the cases of (ClCN)^−^ and (HNC)^−^, contributing 55 and 70% of the total *D*, respectively, while for (HCN)^−^ this contribution is smaller and destabilizing.

Finally, when all contributions are taken into account, the excess electron binding energies calculated for the (HCN)^−^, (HNC)^−^, and (ClCN)^−^ anions are 10, 46, and 22 cm^–1^, respectively, indicating that we are dealing with very weakly bound dipole-bound anions. As demonstrated in Figure 1, the excess electron in each of these anions is highly diffuse and localized well outside the valence region, on the positive side of the molecular dipole, which corresponds to the hydrogen atom (in the case of hydrogen cyanide and its tautomer) or the chlorine atom (in the case of cyanogen chloride). We note that the contours shown in Figure 1 were generated using SCF-level electron densities, and thus their spatial extent reflects the excess electron binding energies obtained at the SCF level (*D* ^*KT*^ + Δ*D*_*ind*_^*SCF*^ in Table 2).

**Figure 1:**
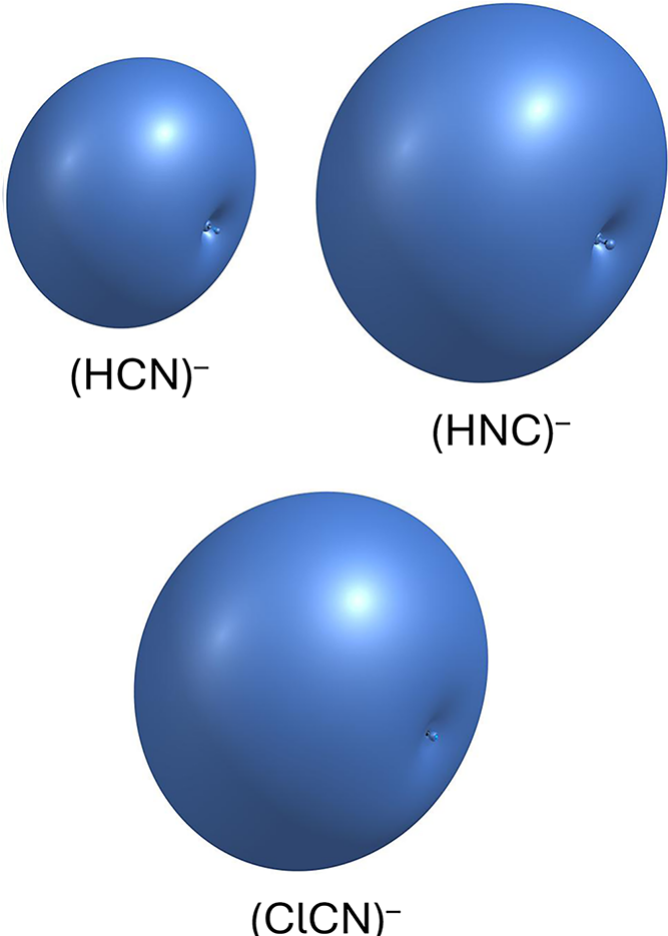
Excess electron density associated with the (HCN)^−^, (HNC)^−^, and (ClCN)^−^ anions. Each contour represents a region containing 50% of the excess electron density.

### 3.2 Dipole-Bound Anions formed by ClCN with Either HCN or HNC: σ-Hole and Hydrogen-Bonded Complexes

#### 3.2.1 Structures of Neutral and Anionic Complexes and Their Relative Energies

The HCN and HNC molecules are polar (see Table 2), and each of them can form two isomers with ClCN, one stabilized by a hydrogen bond and the other by a halogen bond. This results from the fact that the ClCN molecule, in addition to its ability to engage in hydrogen bonding through the negative end of its dipole (the CN group) with hydrogen atoms of other molecules, can also interact via a stabilizing σ-hole interaction with the negative end of another molecule (for instance, with the −CN or −NC group of HCN or HNC, respectively). This is illustrated in Figure 2, which highlights the latter ability by showing a distinct region of positive electrostatic potential near the chlorine atom, corresponding to the positive end of the ClCN molecular dipole and enabling halogen-bond formation through a σ-hole interaction.

**Figure 2:**
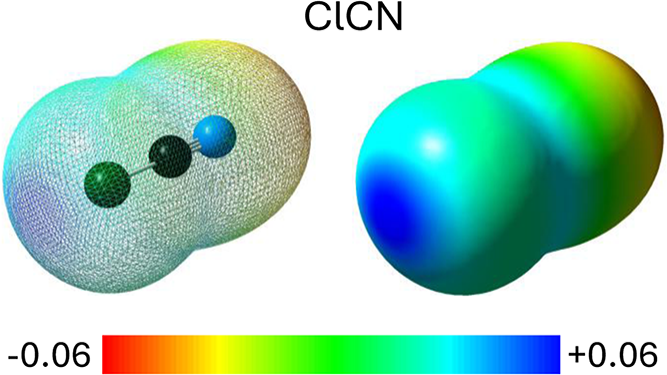
Molecular electrostatic potential map of the isolated neutral ClCN molecule, shown in two views (transparent and opaque). The map is based on the CCSD electron density and plotted on the 0.01 *e*/bohr^3^ isodensity surface. The color scale bar, displayed below the map, indicates electrostatic potential values in atomic units (a.u.), ranging from −0.06 to +0.06.

As shown by our calculations, both HCN and HNC indeed form two such isomers with the ClCN molecule, each exhibiting a linear geometry and C_∞v_ symmetry, both in their neutral and anionic forms (see Figure 3). Formally speaking, all four neutral structures (HCN···ClCN, ClCN···HCN, HNC···ClCN and ClCN···HNC) represent isomers on the potential energy surface of the neutral system, just as their corresponding negatively charged counterparts represent isomers on the anionic potential energy surface. However, it is much more convenient to compare their stabilities within fragment-specific pairs, that is, HCN/ClCN and HNC/ClCN.

**Figure 3:**
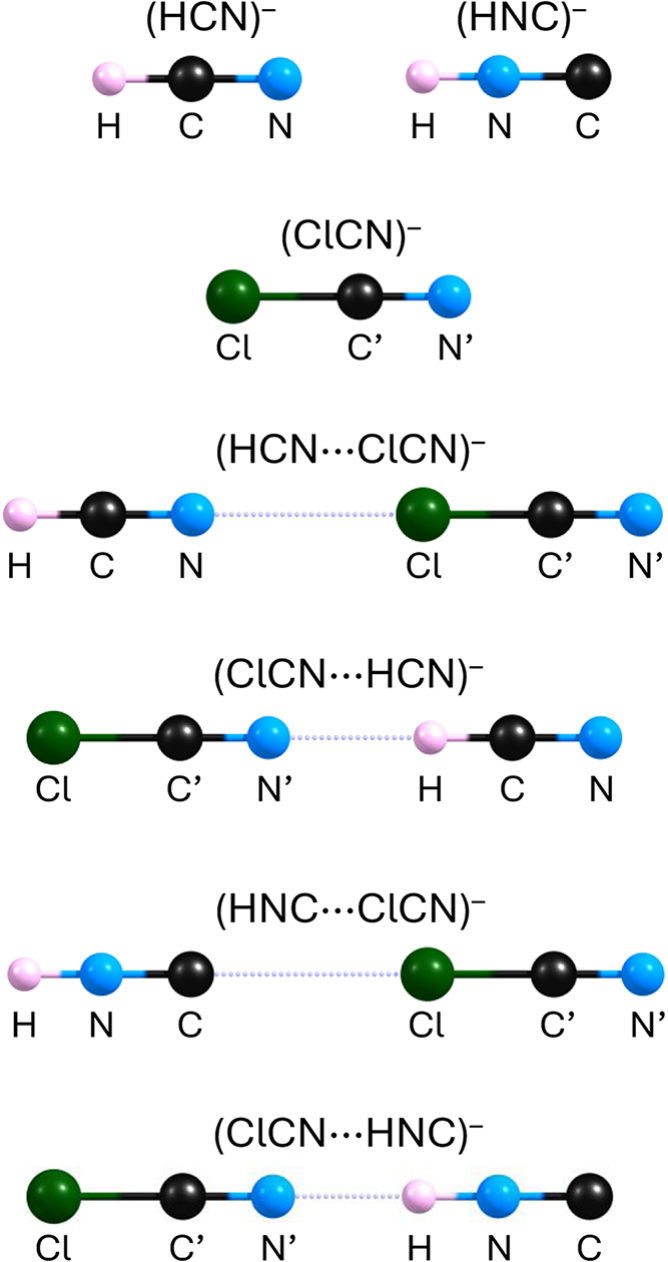
Stationary point structures of the anionic systems discussed in this work. See Table 3 for structural parameters.

Because HCN is significantly more stable than its tautomer HNC, the neutral complexes involving HCN (HCN···ClCN and ClCN···HCN) exhibit considerably lower relative energies (Δ*E*) than those formed with HNC (HNC···ClCN and ClCN···HNC). Specifically, the lowest-energy neutral structure corresponds to the ClCN···HCN complex (Δ*E* = 0.00 kcal/mol) stabilized by a hydrogen bond, while the halogen-bonded HCN···ClCN isomer lies 3.01 kcal/mol higher in energy. The neutral complexes with the HNC moiety display substantially higher relative energies (12.33 and 18.36 kcal/mol for ClCN···HNC and HNC···ClCN, respectively), with the hydrogen-bonded structure again being more stable. In the corresponding anionic systems, the energetic ordering of these four complexes is identical, with the most stable isomer being (ClCN···HCN)^−^ (Δ*E* = 0.00 kcal/mol), followed by (HCN···ClCN)^−^, (ClCN···HNC)^−^, and (HNC···ClCN)^−^, whose relative energies with respect to (ClCN···HCN)^−^ are 4.37, 11.37, and 19.14 kcal/mol, respectively.

As far as structural parameters are concerned, the values collected in Table 3 indicate that the covalent bond lengths within the HCN, HNC, and ClCN fragments are very similar across all complexes. Namely: (i) H–C bond lengths are 1.060 and 1.061 Å in the neutral complexes and 1.061 and 1.064 Å in the anionic ones; (ii) C–N bond lengths in the HCN-based structures are 1.147 and 1.149 Å for both neutral and anionic forms; (iii) H–N bond lengths are 0.993 and 1.004 Å in the neutral complexes and 0.996 and 1.012 Å in the anions; (iv) N–C bond lengths in the HNC-based systems are 1.162 and 1.163 Å for the neutral forms and 1.161 and 1.162 Å for the anionic ones; (v) Cl–C bond lengths span the 1.629–1.636 Å range in the neutral complexes and the 1.635–1.644 Å range in the anions; and (vi) C–N bond lengths within the ClCN fragment (denoted C′-N′ in Figure 3 and Table 3) fall within the 1.149–1.152 Å range for the neutral systems and 1.151–1.153 Å for the anionic complexes.

**Table 3:** Structural Parameters and Harmonic Vibrational Frequencies of Neutral and anionic C_∞v_-Symmetry Stationary Point Structures of HCN···ClCN, ClCN···HCN, HNC···ClCN, and ClCN···HNC^a^

HCN···ClCN/(HCN···ClCN)^−^	
HCN···ClCN	(HCN···ClCN)^−^
*r*(H–C) = 1.060; *r*(C–N) = 1.147;	*r*(H–C) = 1.061; *r*(C–N) = 1.147;
*r*(N···Cl) = 2.995; *r*(Cl–C′) = 1.635; *r*(C′–N′) = 1.152	*r*(N···Cl) = 2.953; *r*(Cl–C′) = 1.635; *r*(C′–N′) = 1.153
ν_1,2_(π) = 52^b^; ν(σ) = 92^c^; ν(π) = 130^d^; ν(π) = 412^e^; ν(σ) = 755^f^; ν(π) = 757^g^; ν(σ) = 2211^h^; ν(σ) = 2337^i^; ν(σ) = 3469^j^	ν_1,2_(π) = 56^b^; ν(σ) = 99^c^; ν(π) = 130^d^; ν(π) = 418^e^; ν(σ) = 755^f^; ν(π) = 769^g^; ν(σ) = 2210; ν(σ) = 2334^i^; ν(σ) = 3449^j^

ClCN···HCN/(ClCN···HCN)^−^	
ClCN···HCN	(ClCN···HCN)^−^
*r*(Cl–C′) = 1.631; *r*(C′–N′) = 1.150;	*r*(Cl–C′) = 1.641; *r*(C′–N′) = 1.152;
*r*(N···H) = 2.167; *r*(H–C) = 1.061; *r*(C–N) = 1.149	*r*(N···H) = 2.102; *r*(H–C) = 1.064; *r*(C–N) = 1.149
ν_1,2_(π) = 53^b^; ν(σ) = 114^b^; ν(π) = 203^d^; ν(π) = 422^e^; ν(σ) = 772^f^; ν(π) = 921^g^; ν(σ) = 2197^h^; ν(σ) = 2358^i^; ν(σ) = 3493^j^	ν_1,2_(π) = 57^b^; ν(σ) = 122^c^; ν(π) = 209^d^; ν(π) = 369^e^; ν(σ) = 672^f^; ν(π) = 949^g^; ν(σ) = 2191^h^; ν(σ) = 2336^i^; ν(σ) = 3435^j^

HNC···ClCN/(HNC···ClCN)^−^	
HNC···ClCN	(HNC···ClCN)^−^
*r*(H–N) = 0.993; *r*(N–C) = 1.162;	*r*(H–N) = 0.996; *r*(N–C) = 1.161;
*r*(C···Cl) = 3.117; *r*(Cl–C′) = 1.636; *r*(C′–N′) = 1.152	*r*(C···Cl) = 3.062; *r*(Cl–C′) = 1.636; *r*(C′–N′) = 1.153
ν_1,2_(π) = 46^b^; ν(σ) = 89^c^; ν(π) = 116^d^; ν(π) = 413^e^; ν(σ) = 752^f^; ν(π) = 527^g^; ν(σ) = 2146^h^; ν(σ) = 2336^j^; ν(σ) = 3868^j^	ν_1,2_(π) = 50^b^; ν(σ) = 97^c^; ν(π) = 118^d^; ν(π) = 420^e^; ν(σ) = 750^f^; ν(π) = 553^g^; ν(σ) = 2154^h^; ν(σ) = 2332^i^; ν(σ) = 3784^j^

ClCN···HNC/(ClCN···HNC)^−^	
ClCN···HNC	(ClCN···HNC)^−^
*r*(Cl–C′) = 1.629; *r*(C′–N′) = 1.149;	*r*(Cl–C′) = 1.644; *r*(C′–N′) = 1.151;
*r*(N···H) = 1.933; *r*(H–N) = 1.004; *r*(N–C) = 1.163	*r*(N···H) = 1.861; *r*(H–N) = 1.012; *r*(N–C) = 1.162
ν_1,2_(π) = 71^b^; ν(σ) = 139^c^; ν(π) = 203^d^; ν(π) = 434^e^; ν(σ) = 778^f^; ν(π) = 880^g^; ν(σ) = 2133^h^; ν(σ) = 2368^i^; ν(σ) = 3646^j^	ν_1,2_(π) = 75^b^; ν(σ) = 156^c^; ν(π) = 210^d^; ν(π) = 350^e^; ν(σ) = 620^f^; ν(π) = 950^g^; ν(σ) = 2136^h^; ν(σ) = 2338^i^; ν(σ) = 3503^j^

a Bond lengths *r* are given in Å, and vibrational frequencies ν are provided in cm^–1^. All results were obtained at the CCSD­(full)/aug-cc-pVTZ+8*s*9*p*4*d* level of theory.

b Intermonomer bending.

c Intermonomer stretching.

d Intermonomer bending.

e Cl–C′-N′ bending.

f Cl–C′-N′ in-phase stretching.

g H–C–N (or H–N–C) bending.

h H–C–N (or H–N–C) in-phase stretching.

i Cl–C′-N′ out-of-phase stretching.

j H–C–N (or H–N–C) out-of-phase stretching.

On the other hand, the intermolecular noncovalent bond lengths (i.e., hydrogen and halogen bonds) show more pronounced differences among the isomers. In the neutral complexes, hydrogen bonds are significantly shorter (2.167 Å in ClCN···HCN and 1.933 Å in ClCN···HNC) than halogen bonds (2.995 Å in HCN···ClCN and 3.117 Å in HNC···ClCN). The same trend is observed in the anionic complexes, where hydrogen bond lengths are 2.102 Å in (ClCN···HCN)^−^ and 1.861 Å in (ClCN···HNC)^−^, while halogen bond lengths are 2.953 Å in (HCN···ClCN)^−^ and 3.062 Å in (HNC···ClCN)^−^ (see Table 3).

Comparing the neutral structures with their corresponding anions enables an assessment of geometric relaxation upon excess electron attachment. As the differences in covalent bond lengths between neutral systems and their corresponding daughter anions are very small (on the order of a few thousandths of an Å), this discussion can be limited to intermolecular separations, namely the hydrogen and halogen bond lengths. The structural parameters presented in Table 3 consistently show that these noncovalent distances are reduced upon anion formation. This shortening is more pronounced in hydrogen bonds (with bond lengths reduced by 0.065–0.072 Å) than in halogen bonds (with reductions of 0.042–0.055 Å). As a result, taking into account the small changes in covalent bond lengths, all anionic structures are slightly more compact than their neutral parents, with total shortening of 0.049 and 0.050 Å for (ClCN···HCN)^−^ and (ClCN···HNC)^−^, and 0.041 and 0.053 Å for (HCN···ClCN)^−^ and (HNC···ClCN)^−^, respectively. Although this effect (∼0.04 to 0.05 Å) is rather small, its consistent direction may be somewhat counterintuitive at first glance, because it is well established that in systems where an excess electron is bound primarily by a dipole potential, the structural changes accompanying anion formation typically enhance the polarity of the neutral molecular host.^1,3–6^ For linear systems such as those studied here, one might therefore expect an elongation rather than a contraction of the complex to increase its dipole moment. In contrast, our results reveal the opposite trend in terms of geometry. Nonetheless, as shown in the following sections, this structural contraction still leads to an increase in the dipole moment of the neutral complex, thereby confirming the general rule of enhanced polarity upon electron attachment. A similar phenomenon was recently reported by Bowen and co-workers, who observed a shortening (by approximately 0.1 Å) of the N → Si dative bond upon excess electron attachment in dipole-bound anions formed by silatranes.^59^

To conclude this part, the geometric relaxation associated with anion formation, despite leading to slightly shorter structures, enhances the dipole moments of the studied complexes and strengthens the noncovalent interactions between the molecular fragments, as will be further discussed below.

#### 3.2.2 Interaction Energies in Neutral and Anionic Complexes

The noncovalent interactions between ClCN and either HCN or HNC not only stabilize the complexes studied here, rendering their existence possible, but also contribute significantly to the enhancement of excess electron binding energies (as will be shown in the following section). Therefore, it is important to discuss these interactions in more detail, beginning with results obtained using the supermolecular approach. In this computational scheme, interaction energies (which can be interpreted, with a sign change, as hydrogen and halogen bond energies) are evaluated as the difference between the energy of the complex and the sum of the energies of its molecular components (i.e., ClCN and either HCN or HNC). It should be noted that in the case of anionic complexes, where the excess electron must be arbitrarily assigned to one of the molecular fragments, we designated the fragment that constitutes the positive pole of the dipole in the neutral host.

The interaction energies derived from the supermolecular approach employing CCSD­(T) energies for the neutral complexes HCN···ClCN, ClCN···HCN, HNC···ClCN, and ClCN···HNC are equal to −4.47, −7.48, −3.98, and −10.02 kcal/mol, respectively. For the corresponding anionic complexes, the interaction energies are −5.64, −9.97, −5.63, and −13.46 kcal/mol, respectively. These values indicate that (i) neutral and anionic hydrogen-bonded complexes are more strongly bound (by 3.01–7.83 kcal/mol) than their corresponding halogen-bonded analogs, (ii) neutral and anionic hydrogen-bonded complexes involving the HNC fragment are more strongly bound (by 2.54–3.49 kcal/mol) than the corresponding complexes involving HCN, (iii) neutral and anionic halogen-bonded complexes involving HCN are slightly more strongly bound (by 0.01–0.49 kcal/mol) than the analogous HNC complexes, and (iv) the attachment of an excess electron, resulting in a charge-assisted complex (either hydrogen- or halogen-bonded), increases the magnitude of the interaction energy by 1.17–3.44 kcal/mol.

A more detailed insight into the interaction energies was obtained by decomposing them into individual contributions using the SAPT approach. Understandably, due to the fundamentally different methodology compared to the supermolecular framework, SAPT results differ quantitatively from the interaction energies presented above, yet they show qualitative consistency.

A few remarks should be made before discussing the SAPT interaction energies for the studied neutral and anionic complexes. The SAPT0 and SAPT2 + 3­(CCD)­δ_MP2_ techniques differ significantly in accuracy, with the latter being more sophisticated. A comparison of the results in Table 4 for the neutral complexes calculated with both methods shows that SAPT0 interaction energies are roughly 1 kcal/mol higher than those obtained at the SAPT2 + 3­(CCD)­δ_MP2_ level, which is consistent with previous studies reporting SAPT0s tendency to overestimate total interactions due to underestimation of the exchange term and overprediction of dispersion and induction contributions.^60,61^ However, since SAPT0 remains the only SAPT method available for open-shell species, we report its results for all neutral and anionic complexes studied in this work.

**Table 4:** SAPT Interaction Energy Components for the Studied Neutral and Anionic Complexes^a^

	interaction energy (total)		individual contributions			
system	SAPT0	SAPT2 + 3(CCD)δ_MP2_	electrostatics	exchange	induction	dispersion
HCN···ClCN	–4.13	(−3.23)	–4.27 (−4.14)	3.16 (3.52)	–0.92 (−0.64)	–2.09 (−1.96)
(HCN···ClCN)^−^	–5.14		–4.82	3.71	–1.75	–2.28
ClCN···HCN	–5.65	(−4.60)	–6.58 (−6.20)	4.65 (5.03)	–1.80 (−1.42)	–1.92 (−2.01)
(ClCN···HCN)^−^	–6.70		–7.31	5.87	–3.08	–2.18
HNC···ClCN	–3.61	(−3.25)	–4.58 (−4.60)	4.04 (4.17)	–1.02 (−0.72)	–2.05 (−2.10)
(HNC···ClCN)^−^	–4.47		–5.20	4.89	–1.89	–2.27
ClCN···HNC	–7.86	(−7.16)	–9.49 (−9.55)	8.09 (8.96)	–3.70 (−3.65)	–2.76 (−2.92)
(ClCN···HNC)^−^	–9.11		–10.70	10.48	–5.69	–3.20

a Values (in kcal/mol) correspond to total interaction energies and their decomposition into electrostatic, exchange, induction, and dispersion contributions. Results obtained at the SAPT0 level are shown explicitly; SAPT2 + 3­(CCD)­δ_MP2_ values (available for neutral systems only) are given in parentheses.

Having noted this limitation, we can proceed to analyze the results presented in Table 4. Regardless of structure, charge, or SAPT level employed, total interaction energies fall into the −3.23 to −9.11 kcal/mol range, which corresponds to the moderate-strength interaction regime.

Nonetheless, the interaction energies calculated for neutral ClCN···HNC and its corresponding dipole-bound anion are significantly higher in absolute value than those obtained for the other systems, being equal to −7.86 (−7.16 at the SAPT2 + 3­(CCD)­δ_MP2_ level) and −9.11 kcal/mol, respectively. This results from the fact that HNC is a much stronger hydrogen bond donor than HCN. Comparing the hydrogen-bonded ClCN···HNC and (ClCN···HNC)^−^ to the halogen-bonded HNC···ClCN and (HNC···ClCN)^−^, we observe much weaker interaction energies (and thus bond strengths) in the latter. These lower bond strengths are due to the chlorine atom’s σ-hole being a less intense electrostatic site than the hydrogen atom in hydrogen bonds. Additionally, the carbon atom in HNC is a relatively weak electron density donor. A similar situation is found in complexes involving the HCN fragment, though the effect is less pronounced, likely because the −CN group in HCN is a much stronger electron density donor than the – NC group in HNC, which enhances the σ-hole interaction in both neutral and anionic HCN···ClCN complexes, as Table 4 affirms.

Examining the changes in interaction energy upon attachment of an excess electron, SAPT0 results show that the total interaction energy increases by approximately 1 kcal/mol on average in all studied complexes. This increase is primarily due to enhanced electrostatic and induction contributions, with the induction term approximately doubling in magnitude, associated with the formation of anions, as these anions can be treated as charge-assisted hydrogen-bonded^62,63^ or halogen- bonded^64,65^ complexes.

To conclude this part, we note that hydrogen-bonded complexes are more strongly bound (in terms of noncovalent interaction energy between ClCN and either HCN or HNC) than halogen-bonded ones. SAPT analysis indicates that this is primarily due to larger electrostatic and induction contributions in hydrogen-bonded systems. As expected, the electrostatic term represents the dominant contribution in all cases. Destabilizing exchange terms also play an important role, especially in hydrogen-bonded and anionic complexes. The transition from neutral to anionic complexes increases all components (both stabilizing contributions and the destabilizing exchange term) of the total interaction energy, resulting in an increase in hydrogen or halogen bond strength by about 1 kcal/mol on average.

#### 3.2.3 Excess Electron Binding Energies in Dipole-Bound (HCN···ClCN)^−^, (ClCN···HCN)^−^, (HNC···ClCN)^−^, and (ClCN···HNC)^−^ Anions

As explained in the preceding sections, both HCN and HNC form two geometrically stable complexes with ClCN: one stabilized by a hydrogen bond and the other by a halogen bond. This results in four distinct complexes, each sufficiently polar to support the formation of a dipole-bound anion. Notably, none of these systems forms a stable valence-bound anion, as verified by our calculations.

We begin our discussion of the electronic stability of these anions by analyzing the polarity of their neutral parent complexes. As expected, the polarity of these neutral systems is significantly larger than that of their isolated building blocks (i.e., HCN, HNC, and ClCN). As indicated by the dipole moments (μ^*CCSD*^) collected in Table 5, the least polar complex is HCN···ClCN (μ^*CCSD*^ = 6.672 D), while the most polar is ClCN···HNC (μ^*CCSD*^ = 7.417 D). These values reflect more than a 2-fold increase in dipole moments resulting from combining ClCN with either HCN or HNC into a single complex. Previous studies have shown that molecules with dipole moments in the 6–7*D* range are capable of forming dipole-bound anions with electron binding energies typically exceeding 400 cm^–1^ (50 meV), and in some cases approaching 1700 cm^–1^ (210 meV).^1,6^ Therefore, substantial electron binding energies can be expected for the anions formed by the complexes studied here.

**Table 5:** Incremental Excess Electron Binding Energies (in cm^–1^) for the Anionic States of HCN···ClCN, ClCN···HCN, HNC···ClCN, and ClCN···HNC Systems, Obtained Using Neutral and Anionic Geometries Optimized at the CCSD­(full)/aug-cc-pVTZ+8*s*9*p*4*d* Theory Level^a^

	(HCN···ClCN)^−^ neutral geometry	(HCN···ClCN)^−^ anionic geometry	(ClCN···HCN)^−^ neutral geometry	(ClCN···HCN)^−^ anionic geometry
*D* ^*KT*^	308	315	321	336
Δ*D*_*ind*_^*SCF*^	31	32	34	36
Δ*D*_*disp*_^*MP*2^	156	160	332	356
Δ*D*_*no‑disp*_^*MP*2^	–116	–119	–113	–121
Δ*D* ^*MP*3^	6	7	–25	–27
Δ*D* ^*MP*4^	15	15	96	105
Δ*D* ^*CCSD*(*T*)^	2	3	192	212
sum (total *D*)	402	413	837	897
μ^*SCF*^	7.099	7.130	7.367	7.427
μ^*MP*2^	6.658	6.688	6.893	6.943
μ^*CCSD*^	6.672	6.702	6.921	6.975

	(HNC···ClCN)^−^ neutral geometry	(HNC···ClCN)^−^ anionic geometry	(ClCN···HNC)^−^ neutral geometry	(ClCN···HNC)^−^ anionic geometry
*D* ^*KT*^	253	264	346	374
Δ*D*_*ind*_^*SCF*^	29	30	41	45
Δ*D*_*disp*_^*MP*2^	170	177	304	340
Δ*D*_*no‑disp*_^*MP*2^	65	67	63	64
Δ*D* ^*MP*3^	–58	–60	–70	–76
Δ*D* ^*MP*4^	60	62	135	153
Δ*D* ^*CCSD*(*T*)^	84	85	315	365
sum (total *D*)	603	625	1134	1265
μ^*SCF*^	6.637	6.683	7.325	7.447
μ^*MP*2^	6.943	6.989	7.627	7.738
μ^*CCSD*^	6.726	6.771	7.417	7.533

a Dipole moments (in Debyes) of the neutral systems, evaluated at both neutral and anionic geometries, were calculated using SCF (μ*^SCF^*), MP2 (μ*^MP2^*), and CCSD (μ*^CCSD^*) electron densities.

As established in earlier studies on dipole-bound anions, the geometrical relaxation upon excess electron attachment is typically driven by a tendency to increase the stability of the anionic system.^1,3–6,23–26^ This pattern is also observed for the complexes examined in this work. Although the linear geometries of the studied systems undergo slight shortening upon excess electron attachment (see Table 3), a comparison of dipole moments for the neutral species calculated at the neutral and anionic equilibrium geometries (Table 5) reveals that the structural relaxation associated with anion formation leads to a noticeable increase in the polarity of the neutral host. Indeed, transitioning from the neutral to the anionic geometry results in a dipole moment increase of 0.030–0.116*D*, with the largest change observed for the hydrogen-bonded ClCN···HNC system.

This increase in the polarity of the neutral host upon electron attachment enhances the electrostatic attraction between the excess electron and the dipole moment of the neutral molecule. As a result, the excess electron is localized closer to the molecular framework, which facilitates stronger induction and dispersion stabilization. Consequently, the excess electron binding energy of the anion increases. This trend is apparent not only when comparing the total *D* values for each anionic complex calculated at both neutral and anionic geometries (see Table 5), but also when analyzing the corresponding partial contributions to *D* within each system. The increase in *D* is relatively small for halogen-bonded anions (HCN···ClCN)^−^ and (HNC···ClCN)^−^ (11 and 22 cm^–1^, respectively), where the polarity of the neutral host changes only slightly upon electron attachment. In contrast, for the hydrogen-bonded anions (ClCN···HCN)^−^ and (ClCN···HNC)^−^, which exhibit a significant increase in host polarity, the *D* enhancement is substantial, 60 and 131 cm^–1^, respectively.

Having discussed both the change in polarity of the neutral host molecule resulting from the geometrical relaxation triggered by excess electron attachment and the impact of this polarity change on the electronic stability of the investigated anions, we now turn to the analysis of the excess electron binding energies characterizing each complex. However, we discuss the total *D* values and the partial contributions to *D* only for the relaxed anionic geometries, as these correspond to the vertical electron detachment energies, which are accessible experimentally.

The Koopmans’ theorem electron binding energy (see *D* ^*KT*^ values in Table 5) amount to 264 and 315 cm^–1^ for the halogen-bonded (HNC···ClCN)^−^ and (HNC···ClCN)^−^ systems, respectively, and to 336 and 374 cm^–1^ for the hydrogen-bonded (ClCN···HCN)^−^ and (ClCN···HNC)^−^ anions. These values correlate with the corresponding μ^*SCF*^ values of the parent neutral species. The induction terms, associated with orbital relaxation, enhance the electronic stability of all anions by 30–45 cm^–1^, which represents 4–8% of the total *D*.

The electron density associated with the excess electron, computed at the SCF level and shown in Figure 4, provides a visual representation of the relative electron binding strength in each system, as predicted by the Hartree–Fock (HF) method. Within the Hartree–Fock framework, where the value of the excess electron binding energy (i.e., *D* ^*KT*^ + Δ*D*_*ind*_^*SCF*^) arises from electrostatic and exchange interactions of the extra electron with the SCF charge distribution of the neutral molecule and includes both the static polarization and the back-polarization effects, the most tightly bound anion is (ClCN···HNC)^−^, as evidenced by its most compact contour, whereas the most weakly bound is (HNC···ClCN)^−^, which exhibits the largest contour. Still, the observed differences in the spatial extent of the excess electron density remain relatively subtle.

**Figure 4:**
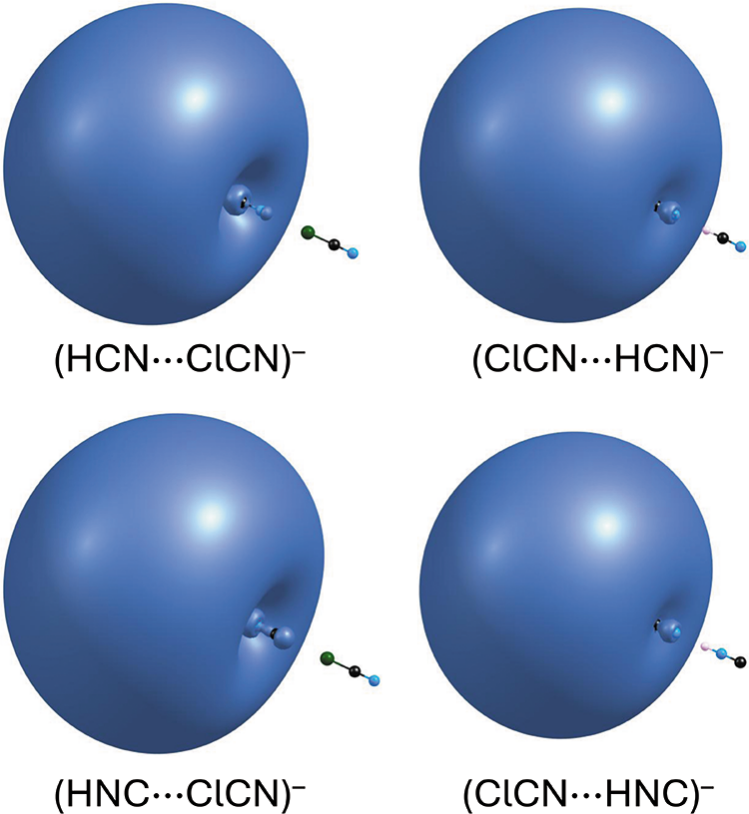
Excess electron density associated with the (HCN···ClCN)^−^, (ClCN···HCN)^−^, (HNC···ClCN)^−^, and (ClCN···HNC)^−^ anions. Each contour represents a region containing 50% of the excess electron density.

In addition, Figure 4 clearly illustrates that the excess electron in each of these anionic complexes is very diffuse and localized outside the valence region, on the positive end of the molecular dipole. This region corresponds to the hydrogen atom in the halogen-bonded systems (HCN···ClCN)^−^ and (HNC···ClCN)^−^, and to the chlorine atom in the hydrogen-bonded systems (ClCN···HCN)^−^ and (ClCN···HNC)^−^.

A comparison of the Δ*D*_*disp*_^*MP*2^ contributions listed in Table 5, which reflect the dynamical correlation between the excess electron and the electrons of the neutral molecule, with the corresponding *D* ^*KT*^ terms reveals a notable difference between halogen-bonded and hydrogen-bonded anions. Specifically, in the hydrogen-bonded systems, the Δ*D*_*disp*_^*MP*2^ terms are comparable in magnitude to the corresponding *D* ^*KT*^ contributions, whereas in the halogen-bonded systems they are clearly smaller, amounting to 51–67% of the respective *D* ^*KT*^ values. However, referencing the Δ*D*_*disp*_^*MP*2^ contributions to the total *D* values alters this perspective: Δ*D*_*disp*_^*MP*2^ accounts for 27 and 40% of the total *D* in the hydrogen-bonded (ClCN···HNC)^−^ and (ClCN···HCN)^−^ systems, respectively, and 28 and 39% in the halogen-bonded (HNC···ClCN)^−^ and (HCN···ClCN)^−^ anions, respectively.

As previously discussed in the context of *D* contributions for (HCN)^−^, (HNC)^−^, and (ClCN)^−^ in earlier sections, electron correlation also modifies the electron density of the neutral molecule, thereby altering its electrostatic interaction with the excess electron. Since this effect emerges for the first time at the second-order Møller–Plesset level (where it is captured by the Δ*D*_*no‑disp*_^*MP*2^ term), the analysis of Δ*D*_*no‑disp*_^*MP*2^ contributions should be conducted in light of the differences between the dipole moment values derived from the SCF electron density (μ^*SCF*^) and those obtained from the MP2 electron density (μ^*MP*2^). For the four anionic complexes considered in this study, a distinct difference can be observed between the systems involving an HCN fragment and those involving an HNC fragment. Specifically, in the former cases, transitioning from an uncorrelated (SCF) to a correlated (MP2) description results in a decrease in the polarity of the neutral host molecule, as evidenced by reductions in dipole moments of 0.442 and 0.484*D* for HCN···ClCN and ClCN···HCN, respectively. This reduction correlates with the negative (and thus destabilizing) Δ*D*_*no‑disp*_^*MP*2^ contributions of −119 and −121 cm^–1^, respectively. In contrast, for complexes containing the HNC fragment, the polarity of the neutral hosts is underestimated at the SCF level, as indicated by higher μ^*MP*2^ values compared to μ^*SCF*^ by 0.306 and 0.291*D* for HNC···ClCN and ClCN···HNC, respectively. As a result, the Δ*D*_*no‑disp*_^*MP*2^ contributions are positive (and thus stabilizing) in the corresponding anions (HNC···ClCN)^−^ and (ClCN···HNC)^−^, amounting to 67 and 64 cm^–1^, respectively (see Table 5).

It is also worth noting at this point that correlation corrections beyond second order no longer significantly modify the dipole moments of the neutral complexes involving the HCN fragment, as the differences between the μ^*CCSD*^ and the corresponding μ^*MP*2^ values are smaller than 0.04*D* in these cases (see Table 5). However, for the complexes containing the HNC fragment, these differences remain non-negligible and amount to approximately −0.20*D*, indicating that, for these latter systems, the electron density distribution of the neutral molecule obtained at the MP2 level is still inadequate, as it overestimates the polarity of the neutral hosts.

The third-order Møller–Plesset (MP3) contributions to *D* are relatively small and destabilizing for all systems except the (HCN···ClCN)^−^ anion, for which the Δ*D* ^*MP*3^ term is stabilizing, although very minor (7 cm^–1^). In contrast, the fourth-order perturbative contributions (denoted as Δ*D* ^*MP*4^ in Table 5) are stabilizing across all anions and account for 4–12% of the total excess electron binding energy in these systems.

As in the case of the HCN^–^, HNC^–^, and ClCN^–^ anions discussed in previous sections, the higher-than-fourth-order contributions to *D*, approximated here by the Δ*D* ^*CCSD*(*T*)^ terms, are found to be significant for all complex anions except (HCN···ClCN)^−^, for which this term is small (3 cm^–1^) and thus nearly negligible, accounting for less than 1% of the total *D*. For the remaining anions, however, the Δ*D* ^*CCSD*(*T*)^ terms are substantial (ranging from 85 to 365 cm^–1^), corresponding to 14–29% of the total excess electron binding energy in these systems. Notably, the Δ*D* ^*CCSD*(*T*)^ terms are substantially larger for the (ClCN···HCN)^−^ and (ClCN···HNC)^−^ anions, whose structures are stabilized by hydrogen bonding, than for the halogen-bonded (HCN···ClCN)^−^ and (HNC···ClCN)^−^ anions (see Table 5).

Finally, when all contributions are taken into account, the excess electron binding energies calculated for the (HCN···ClCN)^−^, (HNC···ClCN)^−^, (ClCN···HCN)^−^, and (ClCN···HNC)^−^ anions amount to 413, 625, 897, and 1265 cm^–1^, respectively. These values indicate that we are dealing with significantly more strongly bound dipole-bound anions than the very weakly bound (HCN)^−^, (HNC)^−^, and (ClCN)^−^ species, whose electronic stabilities do not exceed 50 cm^–1^ (cf. Tables 2, 5). It is worth noting that the anions containing a hydrogen bond in their structure turned out to be approximately twice as strongly bound (in terms of excess electron binding energy) as their counterparts built from the same molecular fragments but stabilized by a σ-hole interaction.

These findings demonstrate that the excess electron binding energy may increase by an order of magnitude upon the formation of a noncovalently bound complex (such as one stabilized by a σ-hole or hydrogen-bond interaction) from molecular units that individually possess only marginal ability to accommodate an excess electron.

## 4 Summary

In summary of the findings presented in this study, we conclude that

i. The polar molecules HCN, HNC, and ClCN form weakly bound dipole-bound anions, with excess electron binding energies of 10, 46, and 22 cm^–1^, respectively.
ii. The ClCN molecule can form two isomeric complexes with either HCN or HNC, one stabilized by a σ-hole interaction (halogen bond) and the other by a hydrogen bond.
iii. The hydrogen-bonded complexes (ClCN···HCN and ClCN···HNC) are more strongly bound than their halogen-bonded counterparts (HCN···ClCN and HNC···ClCN) in terms of the noncovalent interaction energy between ClCN and HCN or HNC.
iv. Upon excess electron attachment, the interaction energies within the complexes increase, leading to a strengthening of the hydrogen or halogen bonds by approximately 1 kcal/mol.
v. The formation of halogen- or hydrogen-bonded complexes leads to a significant increase in the polarity of the resulting systems (by more than a factor of 2), which in turn enables much stronger binding of the excess electron in the resulting dipole-bound anions.
vi. The predicted excess electron binding energies for the halogen-bonded dipole-bound anions (HCN···ClCN)^−^ and (HNC···ClCN)^−^ are 413 and 625 cm^–1^, respectively, while those for the hydrogen-bonded dipole-bound anions (ClCN···HCN)^−^ and (ClCN···HNC)^−^ amount to 897 and 1265 cm^–1^, respectively.
vii. The presence of a hydrogen bond leads to significantly stronger excess electron binding, with such anions being approximately twice as stable as analogous systems stabilized by a σ-hole interaction, despite being constructed from the same molecular fragments.
viii. Our results highlight that excess electron binding can increase by an order of magnitude when a noncovalent complex is formed, whether via hydrogen bonding or σ-hole interaction, from molecular units that, on their own, exhibit only a very limited capacity to bind an excess electron.

## Supplementary Material



## Notes

Cartesian coordinates of all systems (PDF)

## References

[1] Gutowski M., Jordan K. D., Skurski P. (1998). Electronic Structure of Dipole-Bound
Anions. J. Phys. Chem. A.

[2] Simons J. (2008). Molecular
Anions. J. Phys. Chem. A.

[3] Gutowski M., Skurski P., Jordan K. D., Simons J. (1997). Energies of Dipole-Bound
Anionic States. Int. J. Quantum Chem..

[4] Gutowski M., Jordan K. D., Skurski P. (1998). Electronic
Structure of Dipole-Bound
Anions. J. Phys. Chem. A.

[5] Gutowski M., Skurski P., Boldyrev A. I., Simons J., Jordan K. D. (1996). The Contribution
of Electron Correlation to the Stability of Dipole-Bound Anionic States. Phys. Rev. A.

[6] Gutowski M., Skurski P. (1997). Dispersion Stabilization of Solvated
Electrons and
Dipole-Bound Anions. J. Phys. Chem. B.

[7] Gutowski M., Skurski P. (1999). Theoretical Study of
the Quadrupole-Bound Anion (BeO)_2_
^–^. Chem. Phys. Lett..

[8] Marks J., Comita P. B., Brauman J. I. (1985). Threshold resonances
in electron
photodetachment spectra. Structural evidence for dipole-supported
states. J. Am. Chem. Soc..

[9] Mead R. D., Lykke K. R., Lineberger W. C., Marks J., Brauman J. I. (1984). Spectroscopy
and dynamics of the dipole-bound state of acetaldehyde enolate. J. Chem. Phys..

[10] Desfrançois C., Abdoul-Carime H., Khelifa N., Schermann J. P., Brenner V., Millie P. (1995). Dipole binding: An experimental test
for small cluster structure calculations. J.
Chem. Phys..

[11] Mullin A.
S., Murray K. K., Schulz C. P., Szaflarski D. M., Lineberger W. C. (1992). Autodetachment
spectroscopy of vibrationally excited
acetaldehyde enolate anion, CH_2_CHO^–^. Chem. Phys..

[12] Compton R. N., Carman H. S., Desfrançois C., Abdoul-Carime H., Schermann J. P., Hendricks J. H., Lyapustina S. A., Bowen K. H. (1996). On the binding of electrons to nitromethane:
Dipole and valence bound anions. J. Chem. Phys..

[13] Desfrançois C., Abdul-Carime H., Schermann J. P. (1996). Electron attachment to isolated nucleic
acid bases. J. Chem. Phys..

[14] Hendricks J. H., Lyapustina S. A., de Clercq H. L., Snodgrass J. T., Bowen K. H. (1996). Dipole bound, nucleic acid base anions studied via
negative ion photoelectron spectroscopy. J.
Chem. Phys..

[15] Hendricks J. H., de Clercq H. L., Lyapustina S. A., Bowen K. H. (1997). Negative ion
photoelectron spectroscopy of the ground state, dipole-bound
dimeric anion, (HF)_2_
^–^. J. Chem. Phys..

[16] Dessent C. E. H., Kim J., Johnson M. A. (1998). Photochemistry of Halide Ion–Molecule
Clusters: Dipole-Bound Excited States and the Case for Asymmetric
Solvation. Acc. Chem. Res..

[17] Roscioli J. R., Hammer N. I., Johnson M. A. (2006). Infrared
Spectroscopy of Water Cluster
Anions, (H_2_O)_n=3–24_
^–^ in the HOH Bending Region: Persistence of the Double H-Bond Acceptor
(AA) Water Molecule in the Excess Electron Binding Site of the Class
I Isomers. J. Phys. Chem. A.

[18] Asplund M., Koga M., Wu Y. J., Neumark D. M. (2024). Time-resolved photoelectron
spectroscopy of iodide–4-thiouracil cluster: The ππ*
state as a doorway for electron attachment. J. Chem. Phys..

[19] Kim J., Kang D. H., Cheng M., Kim S. K. (2024). Dynamic Interplay
between the Mode-Randomization and Autodetachment of the Dipole-Bound
States of the Anion. J. Phys. Chem. Lett..

[20] Kim J., Kim J., Kim S. K. (2025). Spectroscopy
and Dynamics of the Dipole-Bound States
of ortho-, meta-, and para-Methylphenolate Anions. J. Phys. Chem. A.

[21] Kang J., Brewer E. I., Yuan D.-F., Zhang Y.-R., Wang L.-S. (2025). Photoelectron
Imaging and Photodetachment Spectroscopy for the Cryogenically Cooled
Cyanocyclopentadienide Anion. J. Phys. Chem.
A.

[22] Andersen L. H., Rasmussen A. P., Pedersen H. B., Klinkby N. (2025). Time-Resolved Cryogenic
Action Spectroscopy of Dipole-Bound States in Phenoxide. Phys. Rev. Lett..

[23] Skurski P., Gutowski M., Simons J. (2001). Ab Initio
Electronic Structure of
HCN^–^ and HNC^–^ Dipole-Bound Anions
and a Description of Electron Loss Upon Tautomerization. J. Chem. Phys..

[24] Skurski P., Gutowski M., Simons J. (1999). Theoretical Study of the Dipole-Bound
Anion (HPPH_3_)^−^. J. Chem. Phys..

[25] Skurski P., Gutowski M. (1998). Theoretical Study of the Dipole-Bound Anion (H_2_O···NH_3_)^−^. J. Chem. Phys..

[26] Skurski P., Gutowski M. (1999). Ab Initio Study of a Dipole-Bound Anion (H_2_O···HCl)^−^. J. Chem. Phys..

[27] Oyler N. A., Adamowicz L. (1993). Electron attachment to uracil: theoretical ab initio
study. J. Phys. Chem. A.

[28] Oyler N. A., Adamowicz L. (1994). Theoretical
ab initio calculations of the electron
affinity of thymine. Chem. Phys. Lett..

[29] Roehrig G. H., Oyler N. A., Adamowicz L. (1995). Dipole-Bound
Excess-Electron States
of Adenine Tautomers. A Theoretical ab Initio Study. J. Phys. Chem. A.

[30] Brzeski J., Jordan K. D. (2022). Non-Valence Anions of Pyridine and the Diazines. J. Phys. Chem. A.

[31] Slimak S., Jordan K. D. (2022). Binding of an Electron
by a Finite Fixed Dipole. J. Phys. Chem. Lett..

[32] Lian Y., Xiao L., Li L., Bian L., Xu H., Yan B. (2023). Excited dipole bound
electronic states of potassium iodide anions:
A theoretical perspective. AIP Adv..

[33] Paran G. P., Utku C., Jagau T.-C. (2024). On the performance of second-order
approximate coupled-cluster singles and doubles methods for non-valence
anions. Phys. Chem. Chem. Phys..

[34] Chou Y.-C. (2023). The low-lying
electronic states of ScO_2_ and ScO_2_
^–^: a computational study. Mol. Phys..

[35] Jordan K. D., Wang F. (2003). Theory of Dipole-Bound
Anions. Annu. Rev. Phys.
Chem..

[36] Sawicka A., Skurski P. (2002). Dipole-bound anions supported by charge-transfer interaction:
valence- and dipole-bound anionic states of H_3_N→BF_3_. Chem. Phys..

[37] Sawicka A., Anusiewicz I., Skurski P., Simons J. (2003). Dipole-bound anions
supported by charge-transfer interaction: anionic states of H_n_F_3‑n_N→BH_3_ and H_3_N→BH_n_F_3‑n_ (n = 0,1,2,3). Int. J. Quantum Chem..

[38] Politzer P., Murray J. S., Clark T. (2013). Halogen bonding and other σ-hole
interactions: a perspective. Phys. Chem. Chem.
Phys..

[39] Bartlett R.
J., Purvis G. D. (1978). Many-body perturbation theory, coupled-pair
many-electron theory, and the importance of quadruple excitations
for the correlation problem. Int. J. Quantum
Chem..

[40] Scuseria G. E., Janssen C. L., Schaefer H. F. (1988). An efficient
reformulation of the closed-shell coupled cluster single and double
excitation (CCSD) equations. J. Chem. Phys..

[41] Dunning T. H. (1989). Gaussian basis
sets for use in correlated molecular
calculations. I. The atoms boron through neon and hydrogen. J. Chem. Phys..

[42] Kendall R. A., Dunning T. H., Harrison R. J. (1992). Electron affinities
of the first-row atoms revisited. Systematic basis sets and wave functions. J. Chem. Phys..

[43] Schmidt M. W., Ruedenberg K. (1979). Effective convergence to complete orbital bases and
to the atomic Hartree-Fock limit through systematic sequences of Gaussian
primitives. J. Chem. Phys..

[44] Gutowski M., Simons J. (1990). Double-Rydberg anions:
Ground-state electronic and
geometric stabilities. J. Chem. Phys..

[45] Skurski P., Gutowski M., Simons J. (2000). How to Choose
a One-Electron Basis
Set to Reliably Describe a Dipole-Bound Anion. Int. J. Quantum Chem..

[46] Møller C., Plesset M. S. (1934). Note on an Approximation
Treatment for Many-Electron
Systems. Phys. Rev..

[47] Head-Gordon M., Pople J. A., Frisch M. J. (1988). MP2 energy
evaluation by direct methods. Chem. Phys. Lett..

[48] Frisch M. J., Head-Gordon M., Pople J. A. (1990). A direct MP2 gradient method. Chem. Phys. Lett..

[49] Purvis G. D., Bartlett R. J. (1982). A full coupled-cluster
singles and
doubles model: The inclusion of disconnected triples. J. Chem. Phys..

[50] Koopmans T. (1934). Über
die Zuordnung von Wellenfunktionen und Eigenwerten zu den Einzelnen
Elektronen Eines Atoms. Physica.

[51] Jeziorski B., Moszynski R., Szalewicz K. (1994). Perturbation Theory Approach to Intermolecular
Potential Energy Surfaces of van Der Waals Complexes. Chem. Rev..

[52] Parker T. M., Burns L. A., Parrish R. M., Ryno A. G., Sherrill C. D. (2014). Levels
of Symmetry Adapted Perturbation Theory (SAPT). I. Efficiency and
Performance for Interaction Energies. J. Chem.
Phys..

[53] Lao K. U., Schäffer R., Jansen G., Herbert J. M. (2015). Accurate Description
of Intermolecular Interactions Involving Ions Using Symmetry-Adapted
Perturbation Theory. J. Chem. Theory Comput..

[54] Patkowski K. (2020). Recent Developments
in Symmetry-Adapted Perturbation Theory. WIREs
Comput. Mol. Sci..

[55] Gonthier J. F., Sherrill C. D. (2016). Density-Fitted Open-Shell
Symmetry-Adapted Perturbation
Theory and Application to π-Stacking in Benzene Dimer Cation
and Ionized DNA Base Pair Steps. J. Chem. Phys..

[56] Turney J. M., Simmonett A. C., Parrish R. M., Hohenstein E. G., Evangelista F. A., Fermann J. T., Mintz B. J., Burns L. A., Wilke J. J., Abrams M. L. (2012). Psi4: An Open-Source
Ab Initio Electronic Structure Program. WIREs
Comput. Mol. Sci..

[57] Frisch, M. J. ; Trucks, G. W. ; Schlegel, H. B. ; Scuseria, G. E. ; Robb, M. A. ; Cheeseman, J. R. ; Scalmani, G. ; Barone, V. ; Petersson, G. A. ; Nakatsuji, H. Gaussian 16, Revision C.01; Gaussian, Inc.: Wallingford CT, 2016.

[58] Gutowski M., Skurski P. (1999). Electron binding energies
in linear dipole-bound (HCN)_n_
^–^ (n = 2–5)
anions. Chem. Phys. Lett..

[59] Sidorkin V. F., Belogolova E. F., Doronina E. P., Liu G., Ciborowski S. M., Bowen K. H. (2020). “Outlaw” Dipole-Bound Anions of Intra-Molecular
Complexes. J. Am. Chem. Soc..

[60] Tarek
Ibrahim M., Wait E., Ren P. (2024). Quantum Mechanics Characterization
of Non-Covalent Interaction in Nucleotide Fragments. Molecules.

[61] Schriber, J. ; Wallace, A. ; Cheney, D. ; Sherrill, C. D. Levels of symmetry adapted perturbation theory (SAPT). II. Convergence of interaction energy components. ChemRxiv, 2025, https://chemrxiv.org/engage/chemrxiv/article-details/67fe885f6e70d6fb2e033804, 10.26434/chemrxiv-2023-ftt1v-v2 (accessed June 24, 2025). 40862381

[62] Kochanek S. E., Clymer T. M., Pakkala V. S., Hebert S. P., Reeping K., Firestine S. M., Evanseck J. D. (2015). Intramolecular Charge-Assisted
Hydrogen
Bond Strength in Pseudochair Carboxyphosphate. J. Phys. Chem. B.

[63] Montero M. D. A., Martínez F. A., Aucar G. A. (2019). Magnetic Descriptors
of Hydrogen Bonds in Malonaldehyde and Its Derivatives. Phys. Chem. Chem. Phys..

[64] Domagała M., Lutyńska A., Palusiak M. (2018). Extremely Strong Halogen Bond. The
Case of a Double-Charge-Assisted Halogen Bridge. J. Phys. Chem. A.

[65] Inscoe B., Rathnayake H., Mo Y. (2021). Role of Charge Transfer
in Halogen
Bonding. J. Phys. Chem. A.

