# Supplementary material for: From Fragile to Firm: Reinforcement of Excess Electron Binding in Dipole-Bound Anions through Sigma-Hole and Hydrogen-Bond Interactions
Source: J Phys Chem A. 2025 Sep 2;129(36):8395–406. doi: 10.1021/acs.jpca.5c05103 (PMC12434659; doi:10.1021/acs.jpca.5c05103)
Supplement: Supplementary file 1 [file jp5c05103_si_001.pdf]

Supporting Information

From Fragile to Firm: Reinforcement of Excess Electron Binding in Dipole-Bound Anions through Sigma-Hole and Hydrogen-Bond Interactions

Piotr Skurski[1,2,3], Jakub Brzeski[1,2,*]

[1] *Faculty of Chemistry, University of Gdańsk, Wita Stwosza 63, 80-308 Gdańsk, Poland*

[2] *QSAR Lab Ltd., Trzy Lipy 3, 80-172, Poland*

[3] *Department of Chemistry, University of Utah, Salt Lake City, Utah 84112, U.S.A.*

[*] corresponding author: jakub.brzeski@ug.edu.pl

Table S1. Cartesian coordinates in Å of the studied systems.

HCN			
1	0.000000000	0.000000000	-1.556012000
6	0.000000000	0.000000000	-0.498558000
7	0.000000000	0.000000000	0.649623000
$(HCN)^-$			
1	0.000000000	0.000000000	-1.556014000
6	0.000000000	0.000000000	-0.498547000
7	0.000000000	0.000000000	0.649624000
HNC			
1	0.000000000	0.000000000	1.419568000
7	0.000000000	0.000000000	0.427994000
6	0.000000000	0.000000000	-0.735921000
$(HNC)^-$			
1	0.000000000	0.000000000	-1.419918000
7	0.000000000	0.000000000	-0.427831000
6	0.000000000	0.000000000	0.735789000
ClCN			
17	0.000000000	0.000000000	-0.976983000
6	0.000000000	0.000000000	0.657281000
7	0.000000000	0.000000000	1.809289000
$(ClCN)^-$			
17	0.000000000	0.000000000	-0.977357000
6	0.000000000	0.000000000	0.657623000
7	0.000000000	0.000000000	1.809904000
HCN···ClCN			
1	0.000000000	0.000000000	-4.709478000
6	0.000000000	0.000000000	-3.649296000
7	0.000000000	0.000000000	-2.502235000
17	0.000000000	0.000000000	0.493248000
6	0.000000000	0.000000000	2.128368000
7	0.000000000	0.000000000	3.280783000
$(HCN···ClCN)^-$			
1	0.000000000	0.000000000	-4.681720000
6	0.000000000	0.000000000	-3.620304000
7	0.000000000	0.000000000	-2.473153000
17	0.000000000	0.000000000	0.479794000
6	0.000000000	0.000000000	2.114622000
7	0.000000000	0.000000000	3.267340000
ClCN···HCN			
17	0.000000000	0.000000000	2.735460000
6	0.000000000	0.000000000	1.104619000
7	0.000000000	0.000000000	-0.045749000
1	0.000000000	0.000000000	-2.212799000
6	0.000000000	0.000000000	-3.273565000
7	0.000000000	0.000000000	-4.422300000
$(ClCN···HCN)^-$			
17	0.000000000	0.000000000	-2.722917000
6	0.000000000	0.000000000	-1.081750000
7	0.000000000	0.000000000	0.069846000
1	0.000000000	0.000000000	2.172265000
6	0.000000000	0.000000000	3.236564000
7	0.000000000	0.000000000	4.385645000

HNC···ClCN			
1	0.000000000	0.000000000	-4.712489000
7	0.000000000	0.000000000	-3.719894000
6	0.000000000	0.000000000	-2.557956000
17	0.000000000	0.000000000	0.558779000
6	0.000000000	0.000000000	2.194880000
7	0.000000000	0.000000000	3.347280000
$(HNC\cdots ClCN)^{-}$			
1	0.000000000	0.000000000	-4.677412000
7	0.000000000	0.000000000	-3.681245000
6	0.000000000	0.000000000	-2.520696000
17	0.000000000	0.000000000	0.541082000
6	0.000000000	0.000000000	2.177122000
7	0.000000000	0.000000000	3.329883000
ClCN···HNC			
17	0.000000000	0.000000000	-2.618771000
6	0.000000000	0.000000000	-0.989663000
7	0.000000000	0.000000000	0.159577000
1	0.000000000	0.000000000	2.093055000
7	0.000000000	0.000000000	3.097406000
6	0.000000000	0.000000000	4.260857000
$(ClCN\cdots HNC)^{-}$			
17	0.000000000	0.000000000	-2.607893000
6	0.000000000	0.000000000	-0.963498000
7	0.000000000	0.000000000	0.187284000
1	0.000000000	0.000000000	2.048218000
7	0.000000000	0.000000000	3.060098000
6	0.000000000	0.000000000	4.222546000
